# MRI measurement of the delayed secondary ischaemic injury following endovascular thrombectomy: results from the REPERFUSE-NA1 study

**DOI:** 10.1093/esj/aakag032

**Published:** 2026-04-08

**Authors:** George S Tadros, Joachim Fladt, Meng Wang, Jen Guo, Jacinta L Specht, Ryan A McTaggart, Richard H Swartz, Thalia S Field, Ruchir Shah, Mayank Goyal, Michael D Hill, Andrew M Demchuk, Michael Tymianski, Christopher D d’Esterre, Philip A Barber

**Affiliations:** Calgary Stroke Program, Departments of Clinical Neurosciences, Radiology, and Community Health Sciences, Hotchkiss Brain Institute, Cumming School of Medicine, University of Calgary, Calgary, Canada; Calgary Stroke Program, Departments of Clinical Neurosciences, Radiology, and Community Health Sciences, Hotchkiss Brain Institute, Cumming School of Medicine, University of Calgary, Calgary, Canada; Stroke Center and Department of Neurology, University Hospital Basel and University of Basel, Basel, Switzerland; Calgary Stroke Program, Departments of Clinical Neurosciences, Radiology, and Community Health Sciences, Hotchkiss Brain Institute, Cumming School of Medicine, University of Calgary, Calgary, Canada; Calgary Stroke Program, Departments of Clinical Neurosciences, Radiology, and Community Health Sciences, Hotchkiss Brain Institute, Cumming School of Medicine, University of Calgary, Calgary, Canada; Calgary Stroke Program, Departments of Clinical Neurosciences, Radiology, and Community Health Sciences, Hotchkiss Brain Institute, Cumming School of Medicine, University of Calgary, Calgary, Canada; Department of Neurology and Neurosurgery, Rhode Island Medical Imaging, 593 Eddy St APC 6 Providence, RI 02903, United States; Department of Medical Imaging, University of Toronto, Sunnybrook Health Sciences Centre, Toronto, Canada; Vancouver Stroke Program, University of British Columbia, Vancouver, Canada; UT Erlanger Neurology, Chattanooga, TN, United States; Calgary Stroke Program, Departments of Clinical Neurosciences, Radiology, and Community Health Sciences, Hotchkiss Brain Institute, Cumming School of Medicine, University of Calgary, Calgary, Canada; Calgary Stroke Program, Departments of Clinical Neurosciences, Radiology, and Community Health Sciences, Hotchkiss Brain Institute, Cumming School of Medicine, University of Calgary, Calgary, Canada; Calgary Stroke Program, Departments of Clinical Neurosciences, Radiology, and Community Health Sciences, Hotchkiss Brain Institute, Cumming School of Medicine, University of Calgary, Calgary, Canada; NoNO Inc., Toronto, ON, Canada; Calgary Stroke Program, Departments of Clinical Neurosciences, Radiology, and Community Health Sciences, Hotchkiss Brain Institute, Cumming School of Medicine, University of Calgary, Calgary, Canada; Calgary Stroke Program, Departments of Clinical Neurosciences, Radiology, and Community Health Sciences, Hotchkiss Brain Institute, Cumming School of Medicine, University of Calgary, Calgary, Canada

**Keywords:** acute ischaemic stroke, delayed secondary ischaemic injury, EVT, neuroprotection

## Abstract

**Background:**

Brain injury due to stroke is an important determinant of long-term disability. The acute lesion visible on MRI with DWI underestimates the total burden of the ischaemic injury. The objectives of this study are: (1) quantify the delayed secondary ischaemic injury volume by calculating the whole and regional brain volume loss at 90-days post-stroke and (2) determine whether brain volume loss independently predicted functional outcome at 90 days.

**Methods:**

REPERFUSE-NA1 is a prospective, multisite MRI sub-study of the ESCAPE–NA1 trial (ClinicalTrialGov #NCT02930018), which randomised participants receiving endovascular therapy (EVT) to nerinetide versus placebo. MRI was acquired immediately after therapy (day 1, <5 hours post-EVT) and at 90-days. The primary outcome was change in whole-brain volume between day 1 and 90. Serial MR metrics were used to generate sample size calculations for future neuroprotectant trials.

**Results:**

A total of 43 patients of mean age 65.1 years (SD = 14.9, 51.2% female, median NIHSS 15 [Q1–Q3 = 11–20]) were included. In the entire cohort, there was significant whole-brain volume loss (*P* < .001), ventricular enlargement (*P* < .001), and cortical grey matter (*P* = .001), subcortical white matter (*P* < .001), thalamic (*P* < .001), and hippocampal (*P* < .001) volume loss in the ipsilateral hemisphere. Baseline DWI volume and ipsilateral hemispheric brain atrophy were significant predictors of functional independence, with *P*-values of < .001. There was no significant association between nerinetide treatment and volume changes at 90-days. For a prospective 90-day neuroprotectant trial to demonstrate 50% reduction, 41 patients per group would be needed using ventricular volume change.

**Conclusion:**

This study indicates that whole-brain volume loss is a feasible measurement of delayed secondary ischaemic injury. Future neuroprotectant clinical trials could utilise MR-based markers of delayed ischaemic injury.

## Graphical Abstract

**Graphical Abstract:**
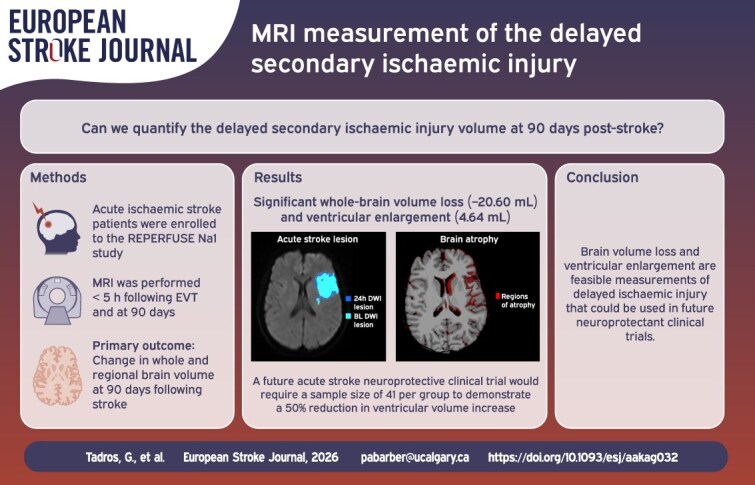

## Introduction

The orthodox concept of ischaemic stroke relates to the compartmental model of “core and penumbra” in which the acutely ischaemic brain comprises both a core of rapidly, irreversibly damaged tissue (“core”) and a less severely hypoperfused region (“penumbra”). The latter is considered the target for treatment because it is potentially salvageable if timely reperfusion occurs.^1–3^ In the absence of reperfusion, the penumbra will inevitably undergo complete tissue infarction, which is termed pan-necrosis.^2^^,^^4^ Depending on the occurrence and timing of early reperfusion, the ischemic penumbra can either recover or progress to pan-necrosis. However, the tissue outcome following ischaemia to the brain has also been observed to be related to selective neuronal loss, which, even if subtle, may impede functional recovery.^5^

SNL occurring within the peri-infarct penumbra cannot be conspicuously detected with MRI.^1^ Delayed injurious mechanisms related to reperfusion will result in tissue loss when there is related selective neuronal injury, axonal degeneration and apoptotic mechanisms. Given that final infarct volume strongly impacts neurological outcomes,^2^ rescuing penumbral tissue after reperfusion can have major clinical relevance, considering there may be significant secondary injury accumulating over hours, days and weeks.^3^ In clinical stroke studies, the measurement of the hyperintense lesion on T2 FLAIR has been conventionally considered as a convenient marker of the final stroke volume at both 30- and 90-days post stroke.^1^ However, if the aim is to take account of the total injury, including delayed secondary injury, this approach is limited by the fact that the necrotic lesion “shrinks” or even disappears over time, and the total injury is then not representative of the slow injurious mechanisms that occur in reperfusion that cause gradual progressive cellular loss.

The eicosapeptide nerinetide (NA1) inhibits the interaction between PSD-95 and the NR2B subunit of the N-methyl-D-aspartate receptor, thereby diminishing neuronal excitotoxicity based on glutamatergic mechanisms associated with acute ischaemic injury.^6^ ESCAPE-NA1 investigated the efficacy of NA1 in reducing disability in patients with large-vessel occlusive strokes undergoing endovascular therapy (EVT). Overall, ESCAPE-NA1 failed to demonstrate a functional benefit of NA1 in stroke patients who received EVT.

In this study, the primary aim was to quantify delayed secondary tissue damage that occurs over hours, days and weeks after EVT in patients treated with NA1 or placebo who were recruited to REPERFUSE-NA1, by measuring reductions in brain volume (brain atrophy), measured after 90-days. In addition, we determined whether brain volume loss independently predicted functional outcome at 90 days. We also calculated sample size estimates that could be used for future neuroprotectant clinical trials based on markers of delayed secondary ischaemic injury.

## Materials and methods

### Study design and patients

REPERFUSE-NA1 was a multisite prospective observational MRI sub-study of the multi-centre, randomized, double-blinded, placebo-controlled ESCAPE-NA1 trial^4^ conducted in Canada (4 sites) and the United States (3 sites).

The inclusion criterion for REPERFUSE-NA1 was the absence of contraindications to MRI, in addition to the ESCAPE-NA1 inclusion criteria. The study was approved by the local ethics board at each site. Written informed consent for study participation was obtained from all patients or their relatives.

### Data acquisition

REPERFUSE-NA1 acquired serial MRI at baseline (<5 hours following EVT), at 24-hours after EVT, and at 90-days. The MRI protocol included DWI and FLAIR sequences at all timepoints. High-resolution T1-weighted imaging was acquired at baseline and 90-days. The detailed standardised MRI protocol has been previously reported.^7^ All sites used 3 T MRI scanners.

Standard assessment of baseline demographic characteristics, medical history and bloodwork was obtained from the ESCAPE-NA1 study. Reperfusion was scored using the expanded Thrombolysis in Cerebral Ischaemia (eTICI) scale. Clinical follow-up was obtained within the ESCAPE-NA1 study at 90-days.

### Image analysis

All imaging data were anonymised, and researchers were blinded to demographic and outcome data while reading scans. Quality assessment was conducted by a trained researcher (G.S.T.) and stroke neurologists (J.F. and P.A.B.) to assess neck/head movement, registration quality and intensity inhomogeneities. Intermediate processing outputs (white matter [WM] hyperintensity volumes and FreeSurfer segmentations and parcellations) were visually inspected for accuracy.^8^ In this study, we used SPM12 to measure the WM hyperintensity volume as well as FreeSurfer version 6.0.0, specifically the longitudinal processing pipeline. Each of these software packages has been previously validated for measurement of WM intensity volumes and whole-brain and regional volume change.^8–11^

The acute infarct was measured on baseline and 24-hour DWI. Final infarct volume was measured using the 90-day FLAIR. Infarct volume on 24-hour FLAIR was also calculated to measure the change in FLAIR lesion volume. Baseline and 90-day whole-brain and regional volumes were measured on T1. White matter hyperintensity (WMH) volumes on FLAIR were automatically measured at baseline using SPM12 software (© 1991, UCL Queen Square Institute of Neurology, London, UK), specifically the 'Lesion segmentation tool’ toolbox applying the “Lesion Prediction Algorithm”.^9^ To exclude lesions associated with acute ischaemia, a mask of the DWI-infarct segmentation was co-registered to the FLAIR images prior to analysis.

Total intracranial volume (TIV) was estimated from baseline T1-weighted images using FreeSurfer^12^ automated skull-stripping and intracranial segmentation. Whole-brain volume was also obtained from the same processing stream (described below). The brain volume/TIV ratio was used as a covariate in all multivariable models to adjust for inter-individual differences in head size and pre-existing global atrophy.

### Delayed secondary ischaemic injury—whole brain and regional volume analysis

Whole-brain tissue volumes, excluding the ventricles, at baseline and 90-days were measured with the longitudinal stream of FreeSurfer 6.0.0. Briefly, this process includes motion correction, removal of non-brain tissue using a hybrid watershed/surface deformation procedure,^13^ automated Talairach transformation, intensity normalisation and atlas registration. Within the longitudinal stream, an unbiased within-subject template space and image is created using robust, inverse consistent registration. The processing steps are then initialized with common information from the within-subject template, significantly increasing reliability and statistical power.^14^

Regional volumes were then calculated using atlas registration as part of the above process.^12^^,^^15^ We specifically focused on the *hemispheric* regional volumes of the cortical grey matter (GM), subcortical WM, subcortical regions of interest including thalamus and hippocampus, as well as the lateral ventricles. These were then classified into ipsilateral and contralateral regional volumes, based on the clinical presentation and infarct location. We calculated the total hemispheric tissue volume, calculated as the sum of the cortical GM, subcortical WM and all subcortical structures segmented in each hemisphere. Quality assessment was conducted manually at various points during analysis to ensure accurate brain extraction, registration and segmentation.

### FLAIR infarct volumes

The evolution of the FLAIR lesion over time was measured at 24-hours and 90-days. Day 90 lesion volume was a priori considered the final infarct tissue volume.^16^^,^^17^ Infarct voxels were defined as those with intensity signal higher than the mean + 2 standard deviations of the signal in the contralateral hemisphere. Manual editing was used to exclude tissue that was unrelated to the infarct.

### DWI infarct volume “growth”

As in previous reports, we measured the early secondary ischaemic injury, defined as the volumetric growth of the ischaemic lesion measured on MR-DWI between baseline and follow-up at 24-hours.^18^

### Statistical analysis

Participant characteristics were described using means with standard deviations (or median and IQR) and frequency distributions. Participant characteristics were compared between placebo and NA1 groups, using Mann–Whitney *U* tests and chi-square tests for continuous and categorical variables, respectively.

Linear mixed-effect (LME) models were first used to assess the effect of timepoint (baseline vs 90-days) on FLAIR lesion volume, as well as delayed secondary ischaemic injury measured by whole-brain volume loss. This was done while controlling for treatment group (NA1 vs placebo). We also examined the change in regional volumes across timepoints, examining the ipsilateral and contralateral volume change separately. Then, a potential 2-way interaction between treatment group and timepoint was explored in separate models. A priori all models were controlled (informed by clinical relevance) for age, sex, thrombolysis, baseline NIHSS, days until follow-up, v/TIV ratio, baseline DWI volume and WMH volume as fixed effects.^4^^,^^18^ The presence of detectable volume change was assessed based on the significance of the effect of time on the volumes examined. Multiple linear regression models were then used to test whether ipsilateral hemispheric atrophy and baseline DWI lesion volume, adjusted for age, predicted functional outcome. Statistical significance was determined at a 2-sided α = 0.05 level. All statistical analyses were performed using SPSS software 29 (IBM Corp., Armonk, NY).

Sample sizes were calculated for stroke patients who received EVT to estimate the number of patients needed for a future randomised controlled trial of a putative neuroprotectant, using absolute volume change of the ventricles, whole-brain, and ROIs between days 1 and 90, and acute DWI growth. We estimated the sample sizes required per arm, for 80% power and 5% Type 1 error rate, using the standard formula^19^:


$$ \mathrm{Sample}\ \mathrm{size}\ \mathrm{per}\ \mathrm{arm}=\frac{{\left(0.84+1.96\right)}^2\left(2{\sigma}^2\right)}{\Delta ^2} $$

where σ^2^ denotes the variance in the outcome. We calculated sample size estimates to detect a reduction in absolute volume change equal to 50% of the change seen in REPERFUSE-NA1 subjects, by setting Δ equal to 0.5 times the estimated mean volumetric change.

## Results

**Figure 1:**
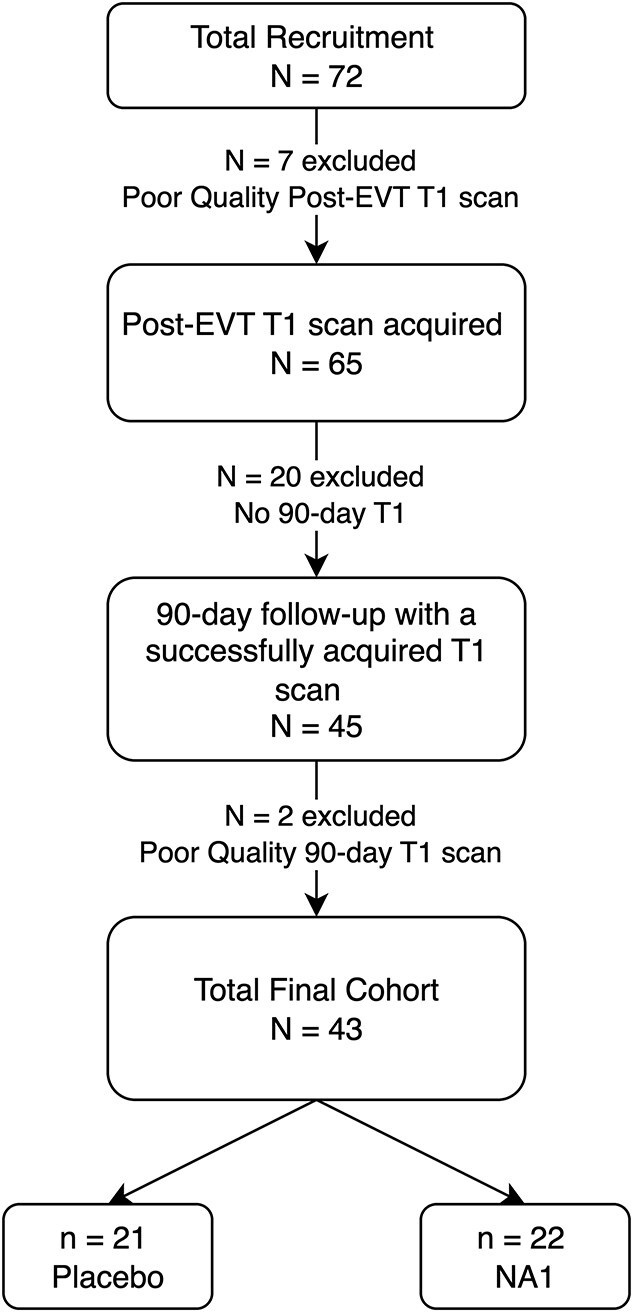
Inclusion and attrition profile.

A total of 43 patients had imaging of sufficient quality and were thus included in the analysis, with 22 patients receiving NA1 and 21 placebo (attrition profile in Figure 1). Demographic and clinical factors for the NA1 and placebo groups are summarised in Table 1. Median FLAIR volume at 24-hours was 15.8 mL (7.9–35.3) in the whole cohort, and at 90-days it was 6.3 mL (2.7–17.8; Tables 2 and 3). There was no statistical difference in median FLAIR volume at 24-hours and 90-days for patients treated with NA1 vs placebo.

**Table 1:** Patient and treatment characteristics.

	Overall cohort (*n* = 43)	Placebo (*n* = 21)	NA1 (*n* = 22)	*P*-value
**Age years, mean standard deviation (SD)**	65.1 (14.9)	65.0 (14.4)	65.2 (15.7)	.874
**Sex Male, *n* (%)**	21 (48.8)	11 (52.4)	10 (45.5)	.650
**Hypertension, *n* (%)**	27 (62.8)	15 (71.4)	12 (54.5)	.252
**Smoking, *n* (%)** ** Current** ** Never** ** Past**	10 (23.3) 22 (51.2) 11 (25.6)	5 (23.8) 10 (47.6) 6 (28.6)	5 (22.7) 12 (54.5) 5 (22.7)	.883
**Hyperlipidaemia, *n* (%)**	22 (51.2)	10 (47.6)	12 (54.5)	.650
**Atrial fibrillation, *n* (%)**	12 (27.9)	5 (23.8)	7 (31.8)	.558
**Diabetes, *n* (%)**	5 (11.6)	5 (23.8)	0 (0)	**.015**
**Congestive heart failure, *n* (%)**	7 (16.3)	0 (0)	7 (31.9)	**.005**
**Past stroke, *n* (%)**	8 (18.6)	3 (14.3)	5 (22.7)	.477
**Witnessed stroke onset, *n* (%)**	29 (67.4)	15 (71.4)	14 (63.6)	.586
**Stroke on awakening, *n* (%)**	6 (14.0)	1 (4.8)	5 (22.7)	.089
**Right hemisphere stroke, *n* (%)**	22 (51.2)	8 (38.1)	14 (63.6)	.094
**NIHSS score, median (Q1–Q3)**	15 (11–20)	16 (13–21)	13 (11–20)	.161
**SBP mmHg, median (Q1–Q3)**	136 (123–148.5)	130 (124–140)	144 (122–151)	.301
**ASPECTS, median (Q1–Q3)**	8 (7–9)	8 (7–9)	8 (7–9)	.871
**Occlusion site ICA, *n* (%)**	15 (34.9)	5 (23.8)	10 (45.5)	.137
**Collaterals good, *n* (%)**	11 (25.6)	4 (19.0)	7 (31.8)	.337
**Alteplase treatment, *n* (%)**	29 (67.4)	14 (66.7)	15 (68.2)	.916
**Interhospital transfer to endovascular** **centre, *n* (%)** **Direct** **Transfer**	31 (72.1) 12 (27.9)	17 (81.0) 4 (19.0)	14 (63.6) 8 (36.4)	.206
**Onset to randomisation mins, median (Q1–Q3)**	113.0 (93.5–186.5)	111 (90–169)	139 (95–250)	.343
**Onset to reperfusion mins, median (Q1–Q3)**	172.5 (120.0–225.0)	165 (117–187.5)	181 (120–298)	.262
**Endovascular centre arrival to arterial access** **or puncture mins, median (Q1–Q3)**	53.0 (43.0–75.0)	53 (42–79)	54 (44–67)	.981
**Study drug start to reperfusion mins, median (Q1–Q3)**	19.0 (5.0–27.0)	17.5 (1.5–26.5)	22 (15–32)	.203
**eTICI** **0–2b** **2c** **3**	18 (41.8) 16 (37.2) 9 (20.9)	7 (33.4) 9 (42.9) 5 (23.8)	11 (50) 7 (31.8) 4 (18.2)	.422
**NIHSS at 24 hours, median (Q1–Q3)**	2.0 (1.0–4.5)	2 (1–5)	2 (1–4)	.931
**3-month mRS 0–2, *n* (%)**	37 (86.0)	17 (81.0)	20 (90.9)	.346

Abbreviations: ASPECTS = Alberta Stroke Program Early CT Score; eTICI = expanded Thrombolysis in Cerebral Infarction; ICA = internal carotid artery; NIHSS = National Institutes of Health Stroke Scale; SBP = systolic blood pressure.

**Table 2:** Infarct volumes, as well as total and region-specific median tissue volumes at baseline (<5 h), 24 hours and 90 days.

	Total cohort	Placebo	NA1	*P*-value
**24-hour FLAIR volume mL, median interquartile range (IQR)**	15.8 (33.4)	15.4 (40.9)	15.8 (21.3)	.952
**90-day FLAIR volume mL, median (IQR)**	6.3 (15.8)	7.3 (23.9)	6.1 (12.3)	.942
**Baseline Tissue volumes mL, median (IQR)**				
***Whole-brain***	1103.8 (194.0)	1093.6 (207.3)	1160.54 (230.7)	.181
***Cortical grey matter***				
***Ipsilateral***	218.0 (43.8)	212.0 (46.0)	218.9 (43.2)	.789
***Contralateral***	217.1 (40.1)	200.5 (44.6)	221.4 (41.1)	.627
***White matter***				
***Ipsilateral***	256.3 (84.0)	245.4 (52.1)	271.8 (104.1)	.076
***Contralateral***	254.3 (82.2)	249.0 (57.2)	276.7 (100.4)	.104
***Thalamus***				
***Ipsilateral***	7.3 (1.7)	7.3 (1.6)	7.4 (1.9)	.343
***Contralateral***	7.2 (1.4)	7.1 (1.6)	7.2 (1.6)	.716
***Hippocampus***				
***Ipsilateral***	3.8 (0.9)	3.8 (1.1)	3.8 (0.6)	.369
***Contralateral***	3.8 (0.8)	3.6 (0.9)	3.9 (0.5)	.437
***Lateral ventricles***	26.1 (22.4)	26.1 (19.5)	28.4 (27.4)	.576
**90-day tissue volumes mL, median (IQR)**				
***Whole-brain***	1094.4 (216.2)	1064.7 (218.7)	1150.6 (213.5)	.114
***Cortical grey matter***				
***Ipsilateral***	204.0 (47.8)	206.9 (50.9)	203.8 (42.7)	.680
***Contralateral***	212.3 (43.5)	212.3 (48.1)	216.9 (41.4)	.752
***White matter***				
***Ipsilateral***	242.6 (81.9)	226.5 (63.5)	269.2 (111.3)	**.037**
***Contralateral***	251.2 (82.8)	247.5 (60.0)	276.3 (113.5)	.114
***Thalamus***				
***Ipsilateral***	6.2 (1.7)	6.5 (1.6)	5.9 (2.2)	.593
***Contralateral***	7.2 (1.4)	7.0 (1.7)	7.2 (1.5)	.662
***Hippocampus***				
***Ipsilateral***	3.8 (1.0)	3.7 (1.3)	3.8 (0.6)	.343
***Contralateral***	3.8 (0.7)	3.7 (1.0)	3.9 (0.3)	.395
***Lateral Ventricles***	29.0 (23.3)	29.0 (22.8)	30.4 (26.5)	.560

**Table 3:** Absolute (90 day—baseline) and percent (of baseline) total and region-specific volume changes.

	Total cohort	Placebo	NA1	*P*-value
**Absolute volume changes mL, median (IQR)**				
***Whole-brain***	−17.5 (23.7)	−17.0 (17.6)	−19.8 (26.6)	.942
***Cortical grey matter***				
***Ipsilateral***	−4.6 (14.4)	−8.5 (12.3)	−2.3 (18.2)	.395
***Contralateral***	0.7 (9.1)	0.7 (9.0)	0.2 (9.5)	.827
***White matter***				
***Ipsilateral***	−6.7 (15.5)	−6.7 (12.2)	−6.8 (16.0)	.528
***Contralateral***	−1.3 (9.8)	−1.4 (8.3)	−1.1 (12.3)	.544
***Thalamus***				
***Ipsilateral***	−0.6 (0.9)	−0.6 (0.9)	−0.8 (1.0)	.627
***Contralateral***	0.00 (0.3481)	−0.04 (0.36)	0.03 (0.3)	.576
***Hippocampus***				
***Ipsilateral***	−0.09 (0.26)	−0.17 (0.20)	−0.06 (0.31)	.481
***Contralateral***	0.02 (0.16)	−0.02 (0.14)	0.05 (0.13)	.174
***Lateral ventricles***	3.2 (4.8)	2.7 (3.7)	3.5 (7.0)	.512
**Percent volume changes %, median (IQR)**				
***Whole-brain***	−1.5 (2.0)	−1.4 (1.7)	−1.6 (2.2)	.771

*P*-values based on Mann–Whitney *U* test.

Linear mixed-effect results for the whole cohort are shown in Table S1. There was a significant decrease in FLAIR lesion volume between 24-hours and 90-days (estimate = -16.34, *P* < .001), after controlling for baseline characteristics. There was also a significant decrease in whole-brain volume over time (estimate = -20.60, *P* < .001). This was accompanied by significant ventricular enlargement (estimate = 4.64, *P* < .001).

Regional volumes for ipsilateral and contralateral regions are shown in Tables 2 and 3. There was significant decrease in cortical GM volume on the ipsilateral side (estimate = -7.02, *P* = .001), while no significant change in contralateral cortical GM volume was observed (estimate = 0.90, *P* = .494). Similarly, subcortical WM volume decreased significantly over time on the ipsilateral side (estimate = -6.66, *P* < .001) but did not change significantly on the contralateral side (estimate = -0.77, *P* = .517). Finally, a similar pattern was seen in the subcortical structures examined. A significant decrease in ipsilateral thalamic and hippocampal volumes was seen (estimate = -0.79 and -0.16; *P* < .00 and .001, respectively) while no significant volume changes were seen on the contralateral side (estimate = -0.01 and -0.00; *P =* .829 and .926, respectively). Ipsilateral hemispheric volume loss exceeded the baseline DWI lesion in 39.5% (*n* = 17) of patients.

To assess the robustness of our statistical approach, we conducted sensitivity analyses using a reduced model retaining key covariates (age and baseline NIHSS score). The results were consistent to those from the primary model (Table S2).

We examined the interaction between treatment group and whole-brain and regional volume changes over time, while controlling for baseline risk factors and covariates. There was no significant effect of treatment group on change in whole-brain and regional volume (Table S3).

In a further analysis, we determined whether baseline DWI volume and ipsilateral hemispheric atrophy predicted functional independence at 90-days (90-day mRS ≤ 2), after adjusting for age. Multiple linear regression results showed that both were significant predictors of functional independence with *P*-values of <.001.

Finally, we calculated sample sizes for future neuroprotectant trials based on previously measured metrics of whole-brain, regional and infarct-specific volume changes between day 1 and 90-day imaging. The sample size calculation results are presented in Table 4. Of the imaging metrics examined, the ventricular volume change (sample size = 41 per group) and the ipsilateral thalamic volume change (sample size = 58) had the smallest sample size needed to detect a 50% reduction of the volume change seen in our cohort.

**Table 4:** Calculated sample size estimates per trial arm for different imaging metrics for a prospective hypothetical 90-day neuroprotectant trial to demonstrate 50% absolute reduction in each metric.

	Sample size per arm (95% CI)
**Ventricular volume change**	41 (39.7–42.0)
**Ipsilateral thalamic volume change**	58 (57.6–58.0)
**Whole-brain volume change**	73 (68.1–78.0)
**Ipsilateral hemispheric volume change**	97 (92.4–100.2)
**DWI lesion growth**	140 (136.5–143.0)
**Ipsilateral subcortical white matter volume change**	185 (183.4–186.6)
**Ipsilateral hippocampal volume change**	204 (203.1–203.2)
**Ipsilateral cortical grey matter volume change**	224 (221.7–225.1)

All sample sizes are calculated per arm (treatment vs placebo).

## Discussion

This study has shown that changes in infarct volumes in all brain compartments can be measured at 90-days after stroke related to large vessel occlusion using serial high-resolution structural MRI. Tissue volume loss, representing delayed secondary brain injury (identified by performing longitudinal volumetric MRI), was shown to occur in the cerebral cortex and subcortical WM and GM, with a substantial portion of the injury occurring beyond the site of the day 1 and 2 DWI, as well as the 90-day FLAIR lesions. While other studies have measured brain atrophy in association of recovery or cognition,^20^ this study is the first to measure rates of whole and regional brain atrophy immediately following EVT over 3-months to our knowledge.

**Figure 2:**
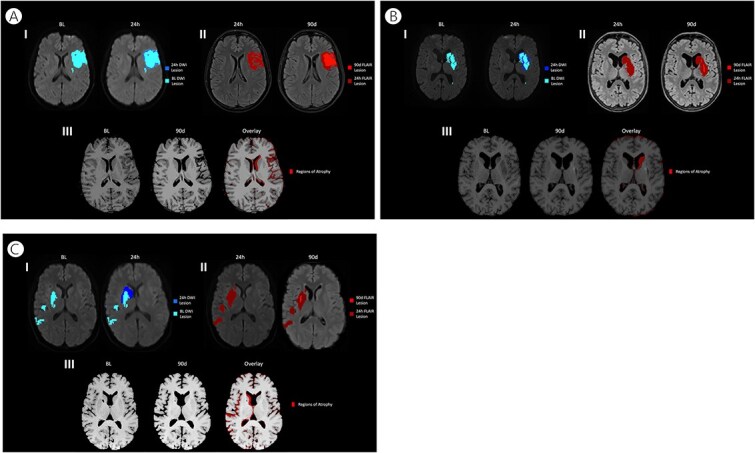
Examples A–C demonstrating the relationship of the acute DWI lesion, 24 hours FLAIR stroke, and the regional brain atrophy over 90 days. For each example, the colour legend indicates I) DWI at baseline (teal) and growth at 24-hours post-stroke (dark blue). II) FLAIR images at 24-hour (red) and 90-days post-stroke (dark red). III) T1-weighted imaging at baseline and 90-days post-stroke, with the regions of brain volume loss shown in red. 2A) Example imaging of a 49-year-old female with left-sided M1 stroke with a baseline NIHSS of 11. She underwent endovascular therapy (EVT) without tPA with a resultant eTICI of 2C and received NA1. 2B) Example imaging of a 66-year-old female with a left-sided M1 stroke with a baseline NIHSS of 12. She underwent EVT with tPA with a resultant eTICI of 3 and received NA1. 2C) Example imaging of a 44-year-old female with a right-sided M1 stroke with a baseline NIHSS of 16. She underwent EVT without tPA with a resultant eTICI of 2B and received placebo.

This study focused on delayed secondary ischaemic injury, particularly selective neuronal loss occurring over days to months after the acute pan-necrotic infarct that is readily detected on DWI and FLAIR. As delayed injury is less easily captured by lesion-based MRI, we have proposed longitudinal brain volume change as a candidate marker of cumulative delayed secondary structural injury to complement conventional infarct volume measures. Whole-brain volumetric analyses showed that there is a concurrent significant reduction in whole-brain tissue volume and ventricular enlargement between baseline and 90-days. The regional brain atrophy was frequently observed to be remote from the DWI lesion and therefore can be concluded to be related the delayed secondary injury, measurable by volume loss, and representing non-necrotic slow injurious mechanisms that occur post-reperfusion. Subsequent regional analyses indicate that significant atrophy occurs in both cortical GM and subcortical WM, even in distant subcortical GM structures, namely the thalamus and hippocampus. This volume loss appears to be confined to the affected stroke hemisphere (example cases are shown in Figure 2).

Conventionally, clinical stroke trials have used FLAIR lesion volume as the outcome of the final injury.^16^ This observation has importance because clinical stroke studies most commonly determine functional outcome at 90 days post stroke, and if concomitant MRI is considered to assess stroke volume, our results suggest that the FLAIR lesion shrinks significantly over 90-days, possibly due to the process of necrotic cell death, with additional contributions from resolving edema. FLAIR volumes were reduced at 90-days in 93% of patients, and there was observed variability in this change, with some FLAIR volume changes being dramatic, with 26% of patients experiencing a ≥70% reduction in FLAIR volume. The FLAIR lesion even vanished completely in 1 patient at 90-days. Therefore, we can conclude that the FLAIR volume is not reliable or a representative marker of the total stroke lesion volume.

In the context of this study, we have confirmed the feasibility of the approach of measuring cerebral and regional brain atrophy over 90-days as a marker of the delayed secondary injury processes that occur following ischaemia reperfusion. This study observes a median atrophy rate of approximately 1.5% across cohort participants in the span of only 90-days. The observed cerebral atrophy commonly extends beyond the identifiable stroke lesion and is localised to the affected ischaemic hemisphere. In our study, this was further corroborated by the fact that 40% of our cohort demonstrated greater ipsilateral hemispheric atrophy than the baseline DWI lesion. Therefore, the atrophy observed cannot be solely explained by pan-necrosis of the baseline DWI lesion, implying the presence of other injury mechanisms that are not fully identified by DWI.

The measurement of brain atrophy rates represents the cumulative ischaemic injury that occurs following ischaemia, which includes both the pan-necrosis and lesion remote SNL, and the delayed injury processes mediated by injurious processes when reperfusion is completely successful, such as inflammation, free radicals and apoptosis.^3^^,^^21^ Numerous modern clinical and imaging studies continue to demonstrate strong associations between infarct volume, subsequent brain atrophy and neurological outcome.^20^^,^^22^ However, preserved brain volume does not necessarily mean preserved function.^23^ Contemporary models of post-stroke disability emphasise that network disconnection and selective neuronal injury, lesion burden, remote neurodegeneration and tissue loss remain fundamental determinants of functional outcome.^24^^,^^25^ A potentially overlooked aspect of post-stroke pathophysiology and cellular injury is the reduced microvascular reperfusion that can occur even with complete recanalization. One primary therapeutic target is the formation of microclots, which may represent the earliest identified pathophysiological manifestation of the reduced reperfusion in the microcirculation when there is successful recanalization. Multiple factors may precipitate the formation of microclots, such as exposure to the extracellular matrix, activation of endothelial cells and involvement of blood clotting factors such as Von Willebrand factor.^26^

MR-defined tissue volume loss is an obvious potential surrogate marker of stroke outcomes. The analysis considers the robustness and the feasibility of such metrics. One potential limitation of measuring brain atrophy in stroke is that when compared to neurodegenerative disease, stroke may contribute to shifts in compartments, particularly in the contralateral side, which may lead to inaccuracies in the volumetric analysis. This might be important when measuring regional volumetric changes specific to anatomical structures. In contrast, volumetric changes that may be less affected by compartmental change include whole brain volume and ventricular volume enlargement because these measurements depend on changes at the brain or ventricular surfaces and are likely to be the most accurate and reproducible; this is perhaps reflective in the smaller calculated sample sizes for whole and ventricular volume change. To safeguard against these potential limitations, intermediate processing outputs require rigorous visual inspection.

We calculated sample sizes for future trials based on all the MR metrics used in our analysis, including DWI growth (as a proposed measure of early secondary ischaemic injury), whole-brain atrophy (as a proposed measure of delayed secondary ischaemic injury), and regional atrophy. Notably, we found that to detect a 50% reduction with 80% power, a total of 41 patients per arm would be required if ventricular volume change is used as a surrogate of the total ischaemic injury. This result has significance for the design of future neuroprotective studies using MRI because the sample sizes needed are potentially much smaller than what might be required for a clinical outcome to detect significant differences. This potentially could be the most cost-effective approach to confirm neuroprotection and inform large, randomised control trials in ischaemic stroke.

The study has limitations. Patient attrition was a significant limitation because of low 90-day attainment. Additionally, not all patients who underwent MRI had a T1w image of sufficient quality to be analysed (Figure 1). Finally, the rate of atrophy measurements of both cerebral hemispheres, cortex, WM and brain regions is affected by both pan-necrosis and selective neuronal injury. Therefore, distinguishing these mechanisms in the context of an overall brain atrophy measurement will still be challenging. However, we have demonstrated that the atrophy can occur in a location remote to the initial DWI lesion, as shown in Figure 2 and Table S1 which might help define selective neuronal injury. Finally, considering the small sample size, we cannot exclude the possibility that the treatment (NA1 or alteplase) had a therapeutic effect on the ischaemic injury, thereby impacting the sample size calculations.

In conclusion, this study has several important findings. First, we show that FLAIR lesion volume decreases significantly in ischaemic stroke patients over 90-days, but there is a concurrent significant volume loss of brain tissue that is restricted to the ipsilateral affected hemisphere. Therefore, using FLAIR volume alone is insufficient to characterise the total burden of brain injury due to the infarct. We confirm that whole-brain volume loss is a feasible measurement of delayed secondary ischaemic injury. We also note that volume loss extends beyond the actual identifiable stroke lesion, into the cortical GM, subcortical WM, and even distant subcortical structures. Finally, we propose the use of several MR-based markers and show their efficacy as potential outcome measures (eg, ventricular volume change) in future clinical trials assessing neuroprotectants for acute ischaemic stroke.

## Supplementary Material

Supplement_RP-NA1_atrophy_ESJ_Second_resubmission_CLEAN_aakag032

## Data Availability

The data that support the findings of this study are available from the corresponding author upon reasonable request.

## Trial registration

Not applicable as this study is not a clinical trial.
