# Supplementary material for: MRI measurement of the delayed secondary ischaemic injury following endovascular thrombectomy: results from the REPERFUSE-NA1 study
Source: Eur Stroke J. 2026 Apr 8;11(4):aakag032. doi: 10.1093/esj/aakag032 (PMC13069884; doi:10.1093/esj/aakag032)
Supplement: Supplement_RP-NA1_atrophy_ESJ_Second_resubmission_CLEAN_aakag032 [file supplement_rp-na1_atrophy_esj_second_resubmission_clean_aakag032.docx]

**Table S1: Linear mixed effect analyses examining the change in FLAIR lesion volume, as well as whole-brain and regional volumes over time (baseline and 90-days), while controlling for group (NA1 vs placebo) and covariates and baseline risk factors (α = 0.05). Full models are shown, including covariates.**

|  | **Estimate** | **Standard Error** | **p-value** |
| --- | --- | --- | --- |
| **FLAIR Lesion** |  |  |  |
| **Time** | -16.13 | 3.54 | **<0.001** |
| Age | 0.17 | 0.22 | 0.457 |
| Baseline NIHSS | 0.12 | 0.53 | 0.819 |
| Sex | 3.97 | 5.49 | 0.474 |
| Thrombolysis | 0.71 | 5.88 | 0.904 |
| WMH Volume | 0.05 | 0.29 | 0.876 |
| Baseline DWI Volume | 1.01 | 0.09 | **<0.001** |
| Days until Follow-up | -0.04 | 0.36 | 0.922 |
| V/TIV Ratio | -7.74 | 34.09 | 0.822 |
|  |  |  |  |
| **Whole brain (excluding ventricles)** |  |  |  |
| **Time** | -20.16 | 3.32 | **<0.001** |
| Age | -1.76 | 1.52 | 0.255 |
| Baseline NIHSS | 1.69 | 3.61 | 0.642 |
| Sex | 129.31 | 37.63 | **0.002** |
| Thrombolysis | -6.72 | 40.34 | 0.869 |
| WMH Volume | 0.89 | 2.01 | 0.661 |
| Baseline DWI Volume | -0.68 | 0.62 | 0.282 |
| Days until Follow-up | 2.77 | 2.45 | 0.266 |
| V/TIV Ratio | 686.72 | 233.86 | **0.006** |
|  |  |  |  |
| **Cortical GM** |  |  |  |
| *Ipsilateral* |  |  |  |
| **Time** | -6.87 | 1.98 | **0.001** |
| Age | -0.95 | 0.29 | **0.002** |
| Baseline NIHSS | -0.14 | 0.68 | 0.840 |
| Sex | 30.81 | 7.08 | **<0.001** |
| Thrombolysis | 19.21 | 7.59 | **0.016** |
| WMH Volume | -0.33 | 0.38 | 0.389 |
| Baseline DWI Volume | -0.15 | 0.12 | 0.196 |
| Days until Follow-up | 0.51 | 0.46 | 0.273 |
| V/TIV Ratio | 7.88 | 44.01 | 0.859 |
|  |  |  |  |
| *Contralateral* |  |  |  |
| **Time** | 0.90 | 1.34 | 0.505 |
| Age | -1.05 | 0.31 | **0.002** |
| Baseline NIHSS | -0.29 | 0.73 | 0.697 |
| Sex | 29.65 | 7.59 | **<0.001** |
| Thrombolysis | 17.13 | 8.13 | **0.043** |
| WMH Volume | -0.35 | 0.40 | 0.397 |
| Baseline DWI Volume | -0.13 | 0.13 | 0.324 |
| Days until Follow-up | 0.29 | 0.49 | 0.561 |
| V/TIV Ratio | -6.01 | 47.16 | 0.899 |
|  |  |  |  |
| **Subcortical WM** |  |  |  |
| *Ipsilateral* |  |  |  |
| **Time** | -6.37 | 1.67 | **<0.001** |
| Age | 0.53 | 0.73 | 0.473 |
| Baseline NIHSS | 1.35 | 1.75 | 0.445 |
| Sex | 23.96 | 18.19 | 0.197 |
| Thrombolysis | -22.69 | 19.50 | 0.253 |
| WMH Volume | 0.76 | 0.97 | 0.436 |
| Baseline DWI Volume | -0.21 | 0.30 | 0.484 |
| Days until Follow-up | 0.68 | 1.19 | 0.570 |
| V/TIV Ratio | 340.54 | 113.03 | **0.005** |
|  |  |  |  |
| *Contralateral* |  |  |  |
| **Time** | -0.65 | 1.16 | 0.579 |
| Age | 0.53 | 0.73 | 0.479 |
| Baseline NIHSS | 1.25 | 1.75 | 0.478 |
| Sex | 26.42 | 18.18 | 0.155 |
| Thrombolysis | -24.44 | 19.49 | 0.218 |
| WMH Volume | 0.75 | 0.97 | 0.445 |
| Baseline DWI Volume | -0.04 | 0.30 | 0.883 |
| Days until Follow-up | 0.46 | 1.19 | 0.698 |
| V/TIV Ratio | 355.63 | 112.99 | **0.003** |
|  |  |  |  |
| **Thalamus** |  |  |  |
| *Ipsilateral* |  |  |  |
| **Time** | -0.79 | 0.12 | **<0.001** |
| Age | -0.02 | 0.01 | 0.157 |
| Baseline NIHSS | -0.02 | 0.03 | 0.525 |
| Sex | 0.64 | 0.31 | **0.043** |
| Thrombolysis | -0.31 | 0.33 | 0.356 |
| WMH Volume | -0.01 | 0.02 | 0.377 |
| Baseline DWI Volume | -0.01 | 0.01 | **0.022** |
| Days until Follow-up | 0.01 | 0.02 | 0.569 |
| V/TIV Ratio | 2.16 | 1.90 | 0.264 |
|  |  |  |  |
| *Contralateral* |  |  |  |
| **Time** | -0.01 | 0.04 | 0.795 |
| Age | -0.04 | 0.01 | **<0.001** |
| Baseline NIHSS | -0.05 | 0.03 | 0.074 |
| Sex | 0.50 | 0.28 | 0.080 |
| Thrombolysis | -0.09 | 0.30 | 0.772 |
| WMH Volume | 0.00 | 0.01 | 0.921 |
| Baseline DWI Volume | 0.00 | 0.00 | 0.573 |
| Days until Follow-up | 0.02 | 0.02 | 0.399 |
| V/TIV Ratio | 2.61 | 1.71 | 0.137 |
|  |  |  |  |
| **Hippocampus** |  |  |  |
| *Ipsilateral* |  |  |  |
| **Time** | -0.16 | 0.04 | **<0.001** |
| Age | -0.02 | 0.01 | **0.024** |
| Baseline NIHSS | -0.00 | 0.02 | 0.782 |
| Sex | 0.20 | 0.18 | 0.263 |
| Thrombolysis | -0.07 | 0.19 | 0.705 |
| WMH Volume | -0.00 | 0.01 | 0.961 |
| Baseline DWI Volume | -0.00 | 0.00 | 0.613 |
| Days until Follow-up | 0.00 | 0.01 | 0.746 |
| V/TIV Ratio | 0.74 | 1.11 | 0.508 |
|  |  |  |  |
| *Contralateral* |  |  |  |
| **Time** | -0.00 | 0.02 | 0.870 |
| Age | -0.02 | 0.01 | **0.011** |
| Baseline NIHSS | -0.00 | 0.02 | 0.815 |
| Sex | 0.20 | 0.16 | 0.211 |
| Thrombolysis | -0.18 | 0.17 | 0.302 |
| WMH Volume | -0.00 | 0.01 | 0.873 |
| Baseline DWI Volume | 0.00 | 0.00 | 0.790 |
| Days until Follow-up | 0.01 | 0.01 | 0.405 |
| V/TIV Ratio | -0.42 | 0.97 | 0.670 |
|  |  |  |  |
| **Lateral Ventricles** |  |  |  |
| **Time** | 4.58 | 0.56 | **<0.001** |
| Age | 0.38 | 0.21 | 0.085 |
| Baseline NIHSS | 0.78 | 0.50 | 0.131 |
| Sex | 9.89 | 5.26 | 0.069 |
| Thrombolysis | -3.92 | 5.63 | 0.491 |
| WMH Volume | 0.26 | 0.28 | 0.354 |
| Baseline DWI Volume | -0.08 | 0.09 | 0.344 |
| Days until Follow-up | -0.33 | 0.34 | 0.344 |
| V/TIV Ratio | -24.85 | 32.67 | 0.452 |

NB: The two timepoints were 24-hour and 90-days for FLAIR lesions and baseline and 90-days for whole-brain and regional volume changes.

**Table S2: Sensitivity analyses with restricted linear mixed effect models with reduced covariates (age and baseline NIHSS), examining the change in FLAIR lesion volume, as well as whole-brain and regional volumes over time (baseline and 90-days). Full models are shown, including covariates.**

|  | **Estimate** | **Standard Error** | **p-value** |
| --- | --- | --- | --- |
| **FLAIR Lesion** |  |  |  |
| **Time** | -16.13 | 3.54 | **<0.001** |
| Age | 0.01 | 0.38 | 0.978 |
| Baseline NIHSS | 1.90 | 1.03 | 0.072 |
|  |  |  |  |
| **Whole brain (excluding ventricles)** |  |  |  |
| **Time** | -20.16 | 3.32 | **<0.001** |
| Age | -1.80 | 1.49 | 0.233 |
| Baseline NIHSS | 0.43 | 4.00 | 0.914 |
|  |  |  |  |
| **Cortical GM** |  |  |  |
| *Ipsilateral* |  |  |  |
| **Time** | -6.87 | 1.98 | **0.001** |
| Age | -1.09 | 0.28 | **<0.001** |
| Baseline NIHSS | -0.58 | 0.76 | 0.454 |
|  |  |  |  |
| *Contralateral* |  |  |  |
| **Time** | 0.90 | 1.34 | 0.505 |
| Age | -1.18 | 0.29 | **<.001** |
| Baseline NIHSS | -0.67 | 0.78 | 0.395 |
|  |  |  |  |
| **Subcortical WM** |  |  |  |
| *Ipsilateral* |  |  |  |
| **Time** | -6.37 | 1.67 | **<0.001** |
| Age | 0.65 | 0.67 | 0.337 |
| Baseline NIHSS | 1.11 | 1.81 | 0.543 |
|  |  |  |  |
| *Contralateral* |  |  |  |
| **Time** | -0.65 | 1.16 | 0.579 |
| Age | 0.61 | 0.68 | 0.370 |
| Baseline NIHSS | 1.30 | 1.82 | 0.479 |
|  |  |  |  |
| **Thalamus** |  |  |  |
| *Ipsilateral* |  |  |  |
| **Time** | -0.79 | 0.12 | **<0.001** |
| Age | -0.02 | 0.01 | **0.035** |
| Baseline NIHSS | -0.04 | 0.03 | 0.134 |
|  |  |  |  |
| *Contralateral* |  |  |  |
| **Time** | -0.01 | 0.04 | 0.795 |
| Age | -0.04 | 0.01 | **<0.001** |
| Baseline NIHSS | -0.04 | 0.03 | 0.081 |
|  |  |  |  |
| **Hippocampus** |  |  |  |
| *Ipsilateral* |  |  |  |
| **Time** | -0.16 | 0.04 | **<0.001** |
| Age | -0.02 | 0.01 | **0.003** |
| Baseline NIHSS | -0.01 | 0.02 | 0.609 |
|  |  |  |  |
| *Contralateral* |  |  |  |
| **Time** | -0.00 | 0.02 | 0.870 |
| Age | -0.02 | 0.01 | **0.001** |
| Baseline NIHSS | -0.00 | 0.01 | 0.876 |
|  |  |  |  |
| **Lateral Ventricles** |  |  |  |
| **Time** | 4.58 | 0.56 | **<0.001** |
| Age | 0.56 | 0.18 | **0.003** |
| Baseline NIHSS | 0.73 | 0.48 | 0.140 |

NB: The two timepoints were 24-hour and 90-days for FLAIR lesions and baseline and 90-days for whole-brain and regional volume changes.

**Table S3: Linear mixed effect analyses examining the effect of the interaction (estimate) between NA1 status and timepoint (baseline vs. Day 90) on whole-brain and regional volume changes while controlling for covariates and baseline risk factors (α = 0.05).**

|  | **Estimate** | **Standard Error** | **p-value** |
| --- | --- | --- | --- |
| **FLAIR Lesion** | -5.48 | 7.12 | 0.446 |
| **Whole Brain (excluding ventricles)** | -4.71 | 6.68 | 0.485 |
| **Cortical GM** |  |  |  |
| *Ipsilateral* | -1.20 | 4.00 | 0.765 |
| *Contralateral* | 0.96 | 2.70 | 0.723 |
| **Subcortical WM** |  |  |  |
| *Ipsilateral* | -4.39 | 3.31 | 0.192 |
| *Contralateral* | -1.12 | 2.34 | 0.634 |
| **Thalamus** |  |  |  |
| *Ipsilateral* | 0.10 | 0.23 | 0.726 |
| *Contralateral* | -0.03 | 0.09 | 0.762 |
| **Hippocampus** |  |  |  |
| *Ipsilateral* | -0.05 | 0.09 | 0.594 |
| *Contralateral* | -0.03 | 0.04 | 0.374 |
|  |  |  |  |
| **Lateral Ventricles** | -0.96 | 1.13 | 0.402 |

NB: All interaction estimates are reported with NA1 being the reference group.
